# Comparison of ruminal microbiota, metabolomics, and milk performance between Montbéliarde×Holstein and Holstein cattle

**DOI:** 10.3389/fvets.2023.1178093

**Published:** 2023-08-02

**Authors:** Haomiao Chang, Xinling Wang, Hanfang Zeng, Yunfei Zhai, Ni Huang, Changjian Wang, Zhaoyu Han

**Affiliations:** College of Animal Science and Technology, Nanjing Agricultural University, Nanjing, China

**Keywords:** Montbéliarde×Holstein, Holstein, milk performance, ruminal microbiota, ruminal metabolomics

## Abstract

Holstein cattle are well known for their high average milk yield but are more susceptible to disease and have lower fecundity than other breeds of cattle. The purpose of this study was to explore the relationship between ruminal metabolites and both milk performance and ruminal microbiota composition as a means of assessing the benefits of crossbreeding Montbéliarde and Holstein cattle. This experiment crossbred Holstein with Montbéliarde cattle, aimed to act as a reference for producing high-quality dairy products and improving the overall efficiency of dairy cattle breeding. Based on similar age, parity and lactation time, 46 cows were selected and divided into two groups (n  =  23 per group) for comparison experiment and fed the same formula: Montbéliarde×Holstein (MH, DIM  =  33.23  ±  5.61 d), Holstein (H, DIM  =  29.27  ±  4.23 d). Dairy herd improvement (DHI) data is an important basis for evaluating the genetic quality of bulls, understanding the quality level of milk, and improving feeding management. We collected the DHI data of these cows in the early lactation, middle lactation and late lactation period of 10  months. The results showed that the average milk production and protein content in Montbéliarde×Holstein were 1.76  kg (34.41  kg to 32.65  kg, *p*  >  0.05) and 0.1% (3.54 to 3.44%, *p*  <  0.05) higher than in Holstein cattle. Moreover, milk from Montbéliarde×Holstein cattle had lesser somatic cell score (1.66 to 2.02) than Holstein cattle (*p*  <  0.01). A total of 10 experimental cattle in early lactation were randomly selected in the two groups (Lactation time  =  92.70  ±  6.81), and ruminal fluid were collected by oral gastric tube. Using 16S rRNA microbial sequencing, we compared the ruminal microbiota composition and found that Montbéliarde×Holstein cattle had a lower abundance of Alphaproteobacteria (*p*  <  0.05) and higher abundance of Selenomonas than Holstein cattle (*p*  <  0.05). These bacteria play roles in protein degradation, nitrogen fixation and lactic acid degradation. The abundance of Succiniclasticum was also greater in Montbéliarde×Holstein cattle (*p*  =  0.053). Through ruminal metabolome analysis, we found that the levels of trans-ferulic acid, pyrrole-2-carboxylic acid, and quinaldic acid were significantly increased in Montbéliarde×Holstein cattle, while that of lathosterol was significantly decreased. The changes in the levels of these metabolites could confer improved antioxidant, anti-inflammatory, and antibacterial activities.

## Introduction

Holstein cattle are well known for their high average milk yield but are more susceptible to disease and have lower fecundity than other breeds of cattle. However, Holstein cattle still hold significant value and purpose. Holstein cattle exhibit excellent traits and characteristics, and despite their health and reproductive challenges, they may still be the most suitable choice for specific market demands or particular farms. During the breeding process, it is necessary to consider market demands, health conditions, and specific advantages in order to develop the best breeding strategy. Hybridization is an important aspect of animal husbandry, through which the advantageous traits of two varieties (lines) can combine to a certain extent in the offspring in a phenomenon known as heterosis (1). It has been reported that heterosis effects can reach more than 6.5% for the productive traits of dairy cattle and more than 10% for traits, such as reproduction, health and lifespan (2). Therefore, the crossbreeding of Holstein cattle is being investigated worldwide to obtain more stable and efficient profits (3). For example, crossbreeding of Fleckvieh and Holstein cattle was found to improve growth performance, slaughter performance, and milk performance in the offspring (4). Several studies have documented the milk composition of Ayrshire, Brown Swiss, Jersey and Holstein hybrid offspring, which had a higher milk yield and fat yield than that of Holstein cattle (5, 6). Some studies have shown that if the breeding goal emphasizes the milk yield, Holstein will still be the preferred breed, and in terms of milk fat and protein yield, it may be better to cross with Juan Shan cattle or Montbéliarde cattle than pure Holstein, and the fertility rate of hybrid cattle is higher (7). Montbéliarde cattle are tall, with small head, deep chest, broad and round hips, deep side belly, developed thigh muscles, long front breast attachment, high and wide rear breast attachment. The average weight of adult cows is 650-800 kg. Compared with Holstein cattle of the same age, Montbéliarde cattle have higher carcass weight, less external fat in the carcass, and more rear leg meat, so the proportion of high-quality muscle is higher (8). In addition, Montbéliarde cattle have the advantages of high disease and stress resistance, high reproductive rates, and long productive lifespan. Therefore, crossbreeding of Montbéliarde and Holstein cattle may improve the quality of dairy products and overcome the low fecundity and poor disease resistance seen in Holstein cattle. Currently, the focus of attention on Montbéliarde×Holstein cattle and Holstein cattle is mainly seen in the comparison of early developmental traits. Montbéliarde×Holstein cattle exhibit better growth trends and evident hybrid vigor (9–11). In terms of milk quality, previous studies found that the protein content of milk and fat content of milk from Montbéliarde×Holstein cattle was significantly higher than that of Holstein cattle (12, 13).

The ruminal microbiota consists of bacteria, fungi, archaea, and protozoa, which degrade and transform feedstuffs, fermented plant proteins, and polysaccharides. This results in the formation of metabolites, including volatile fatty acids, amino acids, and saccharides, which are used to promote microbial growth and reproduction (14). The ruminal microbiota is diverse and complex, influencing production efficiency and affecting the quality of final livestock products. Illumina MiSeq 16S rRNA microbial sequencing is an effective method to analyze microbial communities. Studies have reported a potential relationship between the ruminal microbiota and lactation performance in dairy cows by Illumina MiSeq 16S rRNA microbial sequencing (15). Metabolomic analysis methods include nuclear magnetic resonance (NMR), liquid chromatography–mass spectrometry (LC–MS), and gas chromatography–mass spectrometry (GC–MS). Metabolomics can be used to quantitatively measure metabolic status during lactation and the alterations in metabolites resulting from mastitis (16). Using NMR, Sundekilde (17) found that in milk with a high somatic cell count, levels of lactate, butyrate, isoleucine, acetate, and β-hydroxybutyrate were increased, while levels of hippurate and fumarate decreased, suggesting that changes in milk metabolites can indicate the presence of dairy mastitis. Dervishi (18) used GC–MS for dairy cow serum metabolomics and found that the concentrations of valine, serine, isoleucine, and proline changed before mastitis, and these amino acid changes could be used to indicate udder health. Thus, understanding the composition and function of the microbiota and its metabolites is pivotal for improving the quality of dairy products.

In this study, milk performance, ruminal microbiota composition and ruminal metabolites were compared between Montbéliarde×Holstein and Holstein cattle (same parity) in the same feeding environment. The purpose of this study was to explore the relationship between ruminal metabolites and both milk performance and ruminal microbiota composition as a means of assessing the benefits of crossbreeding Montbéliarde and Holstein cattle. We hypothesize to explain the superior performance of the hybrid cattle by analyzing the correlation of differential metabolites with differential microbial genera and milk performance. In doing so, this study aims to provide a reference for producing high-quality dairy products, improving local dairy cattle varieties and the overall efficiency of dairy cattle breeding.

## Materials and methods

### Animal experiments

The experiment was approved by the Nanjing Agricultural University Institutional Animal Care and Use Committee, and the experiment was conducted at the Xuzhou Weigang Animal Husbandry of Jiangsu Province, China. According to the principle of similar age, parity and lactation time, 23 Montbéliarde×Holstein and 23 Holstein cattle were randomly selected (Table 1) for comparison experiment. These cows are artificially inseminated from January to March, calve from October to December, and the average number of days open was 136 days. The experimental group (Montbéliarde×Holstein, abbreviated as MH) and the control group (Holstein cattle, abbreviated as H) were raised in the same conditions. The cattle in each group had the same total mixed rations (TMR) diet formula (Table 2). The experimental cattle were housed in the same open cowshed and had *ad libitum* access to drinking water. Cattle are fed and milked three times a day. The experimental period was 305 days.

**Table 1:** Cattle selection information.

Items	H	MH	*p-*value
Month age, mo	29.04 ± 0.63	25.58 ± 0.18	0.150
Parity	1.10 ± 0.07	1.00 ± 0.00	0.136
DIM, d	33.23 ± 5.61	29.27 ± 4.23	0.571

**Table 2:** Ingredient of the basal diet (DM^1^ basis).

Ingredients	Content, % of diets	Chemical composition, % of DM	Content, % of DM
Corn silage	43.48	DM	56.08
Whole cottonseed	3.11	ME/(MJ/kg)	16.15
Expand soybean	0.52	Crude Protein	16.31
Corn	16.46	Neutral detergent fiber	33.64
Alfalfa hay	7.87	Acid detergent fiber	21.53
Oat grass	1.66	Ether extract	4.54
Brewers grains	6.21	Ash	7.10
Molasses	2.07	Calcium	0.66
Sugar beet meal	5.18	Phosphorus	0.45
Soybean meal	6.42		
Barley	1.29		
DDGS	1.81		
Fatty powder	0.26		
Premix^2^	2.33		
Yeast compound	0.83		
Sodium bicarbonate	0.52		
合计 Total	100		

1 DM, dry matter.

2 Provided as per kilogram of premix: VA 60KIU, VD 8KIU, VE 300 mg, Cu 300 mg, Zn 1,100 mg, Fe 620 mg, Mn 430 mg, I 8 mg, Co 6 mg, Se 6 mg.

### Sample collection and determination

During the experimental period, DHI data for 10 months was provided by the farm which was measured according to the standard process. The experimental cattle went through three stages: early lactation (DIM = 22 ~ 100d), middle lactation (DIM = 101 ~ 200d) and late lactation (DIM = 201 ~ 305d). Monthly DHI data of the experimental cattle were collected for milk production and milk composition analysis. According to the DHI monitoring and sampling requirements, the milk samples of two groups were collected, and the milk composition of the mixed milk samples (4:3:3) in the morning, middle and evening of each cow for 1 day was determined (19). Milk fat (%), milk protein (%), milk lactose (%), milk total solid (%), milk somatic cell count (×10^4^·mL^−1^), and milk urea nitrogen (mg·dL^−1^) were determined by DHI online detection system (Bentley NexGen FCM-FTS, United States).

A total of 10 experimental cattle in early lactation were randomly selected in the two groups (n = 5 per group, DIM = 92.70 ± 6.81 d). All of the samples were obtained on the same day, and all cows were sampled only once. Ruminal fluid samples were collected by oral stomach tube after 1–2 h of feeding. Each cow collected 100 mL of ruminal fluid, which is filtered through 4 layers of gauze, and then divided into different volume collection tubes. The ruminal fluid was stored at −80°C for ruminal microbiota and metabolomic determination.

### Illumina MiSeq 16S rRNA microbial sequencing

Total genome DNA from samples was extracted using cetyltriethylammnonium bromide (CTAB) method (20). DNA concentration and purity was monitored on 1% agarose gels. According to the concentration, DNA was diluted to 1 ng/μL using sterile water. The V3-V4 region of the 16S rRNA gene was amplified by applying a primer pair of 515F (5’-GTGCCAGCMGCCGCGGTAA-3′)/806R (5’-GGACTACHVGGGTWTCTAAT −3′). All PCR reactions were carried out with 15 μL of Phusion High-Fidelity PCR Master Mix (New England Biolabs). Two micrometer of forward and reverse primers, and about 10 ng template DNA. Thermal cycling consisted of initial denaturation at 98°C for 1 min, followed by 30 cycles of denaturation at 98°C for 10 s, annealing at 50°C for 30 s, and elongation at 72°C for 30 s. Finally 72°C for 5 min. Subsequent sequencing work was handed over to PANOMIX (Suzhou, China).

Bioinformatics analysis was performed using FLASH version 1.2.7 and Quantitative Microbial Ecological Insights (QIIME) version 1.9.1. Sequences analysis were performed by Uparse software (Uparse v7.0.1001)^1^ (21). Sequences with ≥97% similarity were assigned to the same operational taxonomic unit (OTU). For each representative sequence, the Silva Database^2^ (22) was used based on Mothur algorithm to annotate taxonomic information. In order to study phylogenetic relationship of different OTUs, and the difference of the dominant species in different samples (groups), multiple sequence alignment were conducted using the MUSCLE software (Version 3.8.31)^3^ (23). OTUs abundance information were normalized using a standard sequence number corresponding to the sample with the least sequences. Subsequent analysis of alpha diversity and beta diversity were all performed basing on this output normalized data. Alpha diversity is applied in analyzing complexity of species diversity for a sample through 5 indices, including the number of visually observed species (Observed-species), diversity indices (Shannon and Simpson), and richness index (Chao1 and ACE). All this indices were calculated with QIIME (Version 1.9.1). Beta diversity analysis was used to evaluate differences of samples in species complexity, Beta diversity on both weighted and unweighted unifrac were calculated by QIIME software (Version 1.9.1). Principal Coordinate Analysis (PCoA) was performed to get principal coordinates and visualize from complex, multidimensional data, and which was displayed by ade4 package and ggplot2 package in R software (Version 2.15.3).

### LC–MS analysis

Samples were thawed at 4 oC, vortexed for 1 min after thawing, mixed evenly, and transferred into a 2 mL centrifuge tube. Added 400 μL methanol solution (20°C) and vortexed for 1 min. Centrifuge at 12000 rpm at 4°C for 10 min, took all the supernatant, transferred it to a new 2 mL centrifuge tube and concentrated it to dry, added 150 μL of 2-chloro-L-phenylalanine (4 ppm) prepared with 80% methanol water accurately. The solution (stored at 4°C) was used to reconstitute the sample, and the supernatant was filtered through a 0.22 μm membrane, and the filtrate was added to the detection bottle for LC–MS detection (24).

The LC analysis was performed on a Vanquish UHPLC System (Thermo Fisher Scientific, United States). Chromatography was carried out with an ACQUITY UPLC HSS T3 (150 × 2.1 mm, 1.8 μm) (Waters, Milford, MA, USA). The column maintained at 40°C. The flow rate and injection volume were set at 0.25 mL/min and 2 μL, respectively. For LC-ESI (+)-MS analysis, the mobile phases consisted of (C) 0.1% formic acid in acetonitrile (v/v) and (D) 0.1% formic acid in water (v/v). Separation was conducted under the following gradient: 0–1 min, 2% C. 1–9 min, 2–50% C. 9–12 min, 50–98% C. 12–13.5 min, 98% C. 13.5–14 min, 98–2% C. 14–20 min, 2% C. For LC-ESI (−)-MS analysis, the analytes was carried out with (A) acetonitrile and (B) ammonium formate (5 m*M*). Separation was conducted under the following gradient: 0–1 min, 2%A. 1–9 min, 2-50%A. 9–12 min, 50–98%A. 12–13.5 min, 98%A. 13.5–14 min, 98–2%A. 14–17 min, 2%A (25). Mass spectrometric detection of metabolites was performed on Q Exactive HF-X(Thermo Fisher Scientific, United States) with ESI ion source. Simultaneous MS1 and MS/MS (Full MS-ddMS2 mode, data-dependent MS/MS) acquisition was used. The parameters were as follows: sheath gas pressure, 30 arb, aux gas flow, 10 arb, spray voltage, 3.50 kV and − 2.50 kV for ESI(+) and ESI(−), respectively, capillary temperature, 325°C, MS1 range, m/z 81–1,000, MS1 resolving power, 60,000 FWHM, number of data dependent scans per cycle, 8. MS/MS resolving power, 15,000 FWHM, normalized collision energy, 30%, dynamic exclusion time, automatic (26).

The original mass spectrometry offline file was converted into mzXML file format by msconvert tool in proteowizard software package (v3.0.8789) (27). Rxcms software package (28) is used for peak detection, peak filtering and peak alignment to obtain the quantitative list of substances. The parameters are BW = 2, PPM = 15, peakwidth = C (5, 29), mzwidth = 0.015, mzdiff = 0.01, and method = ‘centwave. The public databases HMDB (30), massbank (29), LipidMaps (31), mzcloud (32), KEGG (33) and self-built substance library were used for substance identification, and the parameters were set as ppm < 30 ppm. The LOESS (34) signal correction method based on quality control (QC) samples realizes data correction and eliminates system error. Substances with RSD > 30% in QC samples were filtered out in data quality control.

### Statistical analysis

The milk somatic cell count (SCC) belongs to the skewed distribution, which should be replaced by somatic cell score (SCS) in the analysis of variance (35). The experiment reference to somatic cell count data transformation, the cows to improve planning committee to determine the SCS = $\log_{2}\left(\mathrm{SCC}/100000\right)+3\;$ (36) as a formula to calculate. Statistical analysis was performed in IBM SPSS Statistics 26 (SPSS Inc., Chicago, IL, United States). DHI data were subjected to repeated measures ANOVA. Breed, lactation time, and their interaction were considered as fixed effects and cow as the random effect. DHI data and physiological parameters results were expressed as means ± standard error, and significance was expressed as *p-*value <0.05. Principal Co-ordinates Analysis was used to detect differences between the microbial communities from different experimental groups. T-test was used to identify phylum and genus-level differences in microbes. Metabolites with variable influence on projection (VIP) values larger than 1.0 and *p*-values from a two-tailed Student’s *t*-test <0.05 were considered differential metabolites. Correlation analyses were performed using Pearson’s correlation coefficients obtained for the differential metabolites with differential microbial genera and milk performance.

## Results

### Comparison of milk performance between Montbéliarde×Holstein and Holstein cattle

**Figure 1:**
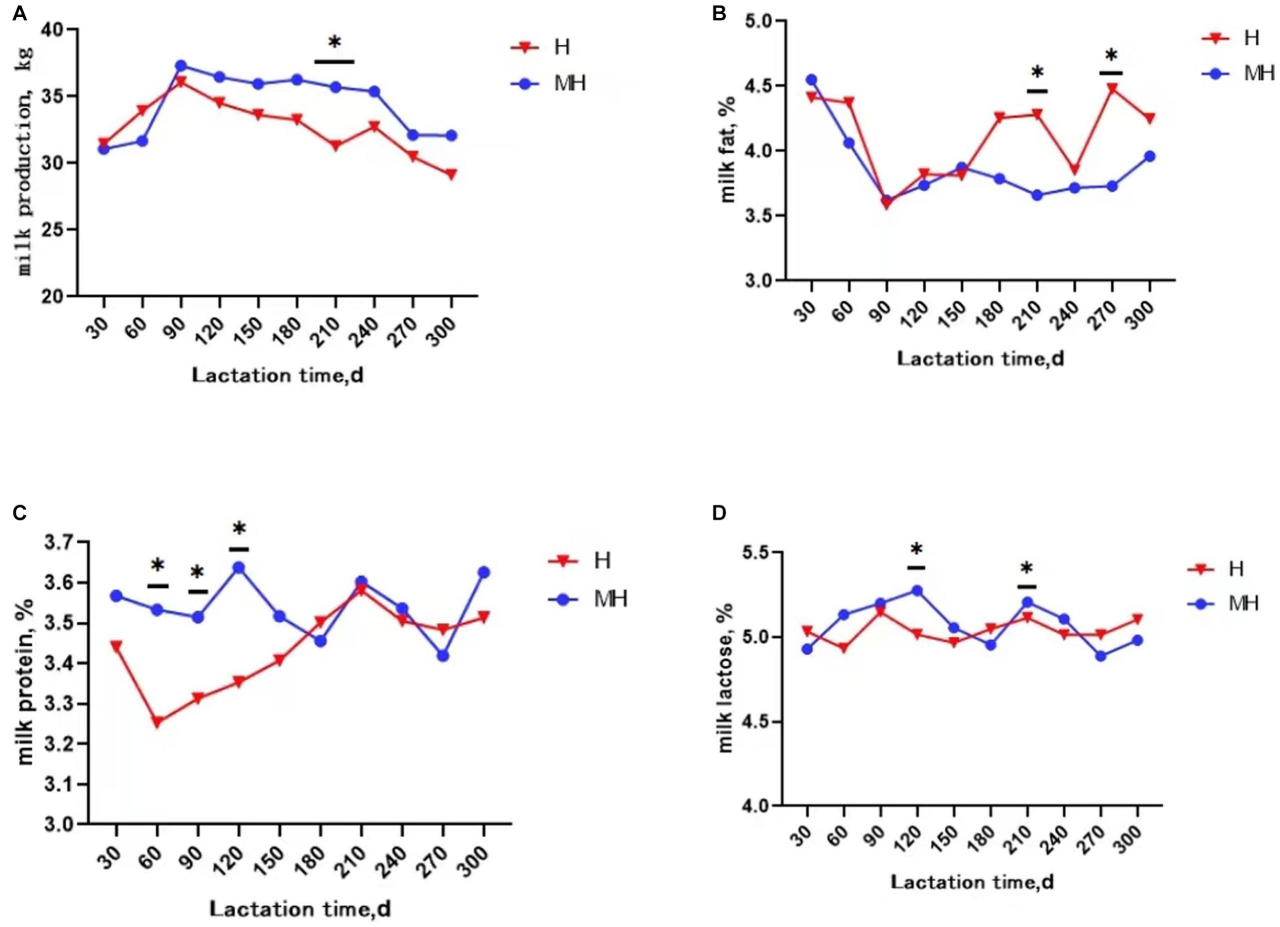
Change curve of milk performance during lactation. * indicates significant differences at *p* < 0.05 level. Milk production **(A)**; milk fat **(B)**; milk protein **(C)**; milk lactose **(D)**.

**Figure 2:**
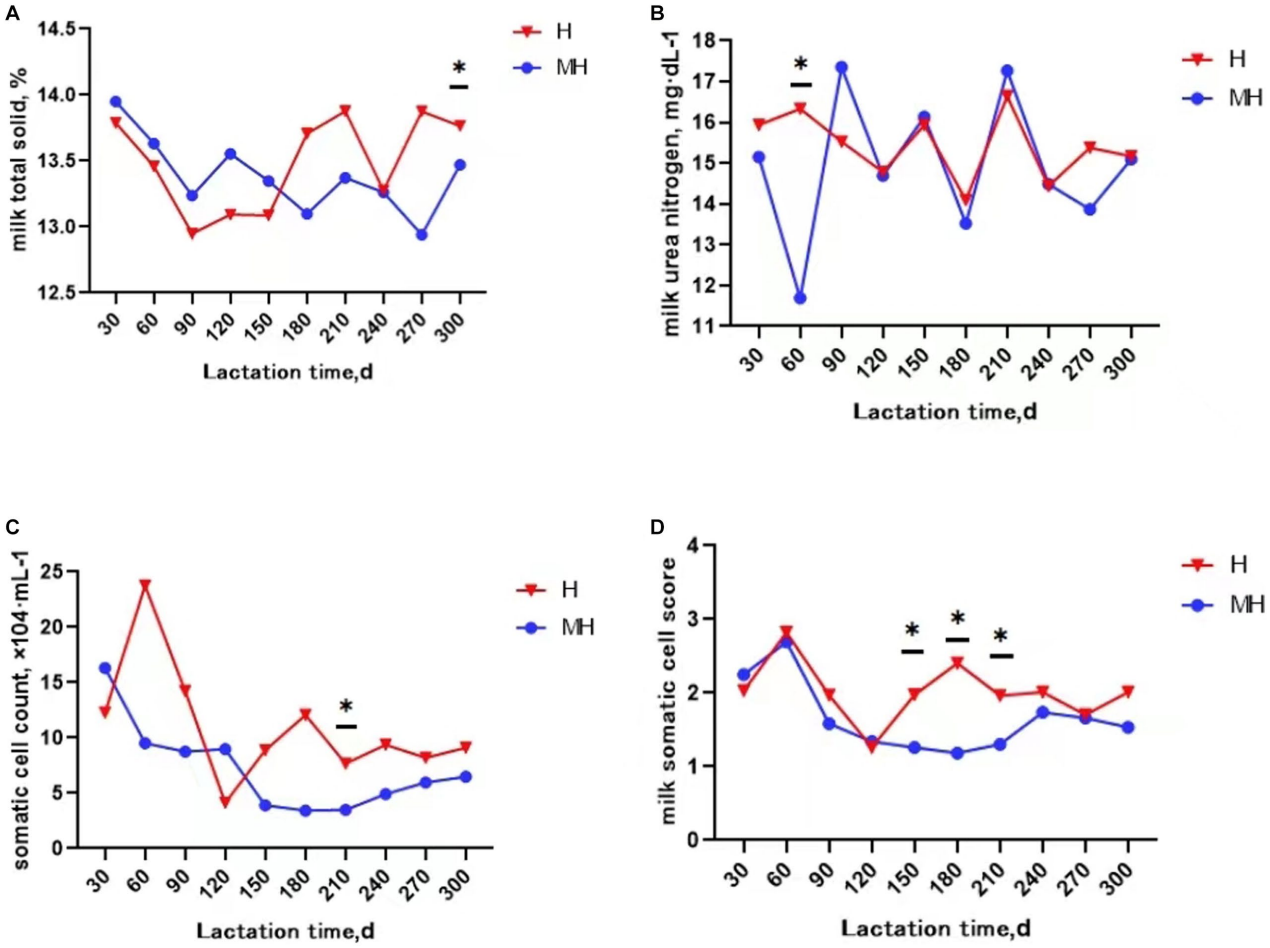
Change curve of milk composition during lactation. * indicates significant differences at *p* < 0.05 level. Milk total solid **(A)**; milk urea nitrogen **(B)**; somatic cell count **(C)**; milk somatic cell score **(D)**.

It can be seen from Table 3 that there was no significant interaction effect between breed and lactation time on daily average milk production and milk composition (*p* > 0.05). Milk protein and milk somatic cell score were significantly affected by breed (*p* < 0.05). The average milk protein content of Montbéliarde×Holstein cattle were 0.1% higher than that of Holstein cattle (*p* < 0.05). The somatic cell score (1.66 to 2.02) were significantly lower in Montbéliarde×Holstein cattle than Holstein cattle. The average milk production and milk composition were significantly affected by lactation time (*p* < 0.05). The average milk production of Montbéliarde×Holstein cattle was significantly higher than that of Holstein cattle at 210d of lactation (*p* < 0.05) (Figure 1A). The average milk fat of Montbéliarde×Holstein cattle was significantly lower than that of Holstein cattle at 210d and 270d of lactation (*p* < 0.05) (Figure 1B). The average milk protein of Montbéliarde×Holstein cattle was significantly higher than that of Holstein cattle at 60d, 90d and 120d of lactation (*p* < 0.05) (Figure 1C). The average milk lactose of Montbéliarde×Holstein cattle at 120d and 210d of lactation was significantly higher than that of Holstein cattle (*p* < 0.05) (Figure 1D). The average milk total solid of Montbéliarde×Holstein cattle at 300d of lactation (Figure 2A), average milk urea nitrogen at 60d of lactation (Figure 2B), and average milk somatic cell count at 210d of lactation (Figure 2C) were all significantly lower than those of Holstein cows (*p* < 0.05). The milk somatic cell score of Montbéliarde×Holste cattle was significantly lower than that of Holstein cattle at 150d, 180d and 210d of lactation (*p* < 0.05; Figure 2D).

**Table 3:** Comparison of milk performance between Montbéliarde×Holstein and Holstein Cattle.

Items	H	MH	*p-*value		
			Breed	Lactation time	Breed × Lactation time
Milk production, kg	32.65 ± 1.07	34.41 ± 1.07	0.252	<0.001	0.377
Milk fat, %	4.11 ± 0.10	3.87 ± 0.10	0.101	0.002	0.107
Milk protein, %	3.44 ± 0.04	3.54 ± 0.04	0.046	<0.001	0.142
Milk lactose, %	5.04 ± 0.03	5.08 ± 0.03	0.358	0.027	0.074
Milk total solid, %	13.57 ± 0.20	14.10 ± 0.20	0.163	0.040	0.247
Milk urea nitrogen, mg·dL^−1^	15.43 ± 0.61	14.93 ± 0.61	0.565	<0.001	0.102
Somatic cell count, ×10^4^·mL^−1^	10.98 ± 1.44	7.18 ± 1.44	0.070	0.042	0.670
Milk somatic cell score	2.02 ± 0.12	1.66 ± 0.12	0.036	0.001	0.529

### Comparison of ruminal microbiota composition between Montbéliarde×Holstein and Holstein cattle

**Figure 3:**
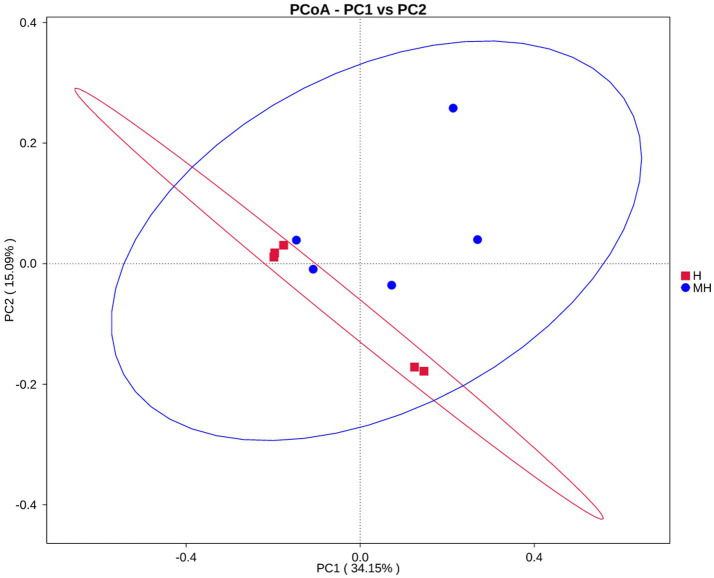
PCoA analysis of rumen bacteria in Montbéliade×Holstein and Holstein Cattle.

**Figure 4:**
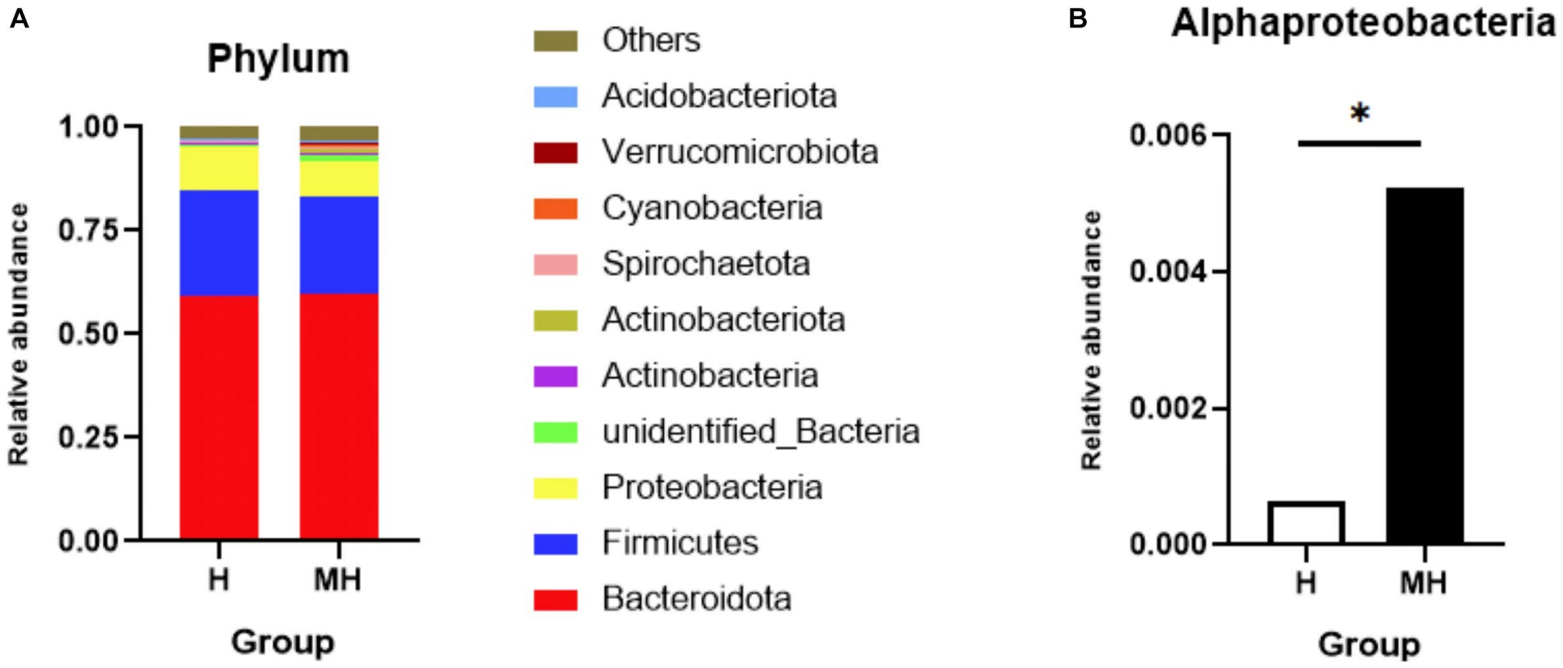
Comparison of phylum level of rumen bacteria between Montbéliade×Holstein and Holstein Cattle. * indicates significant differences at *p* < 0.05 level. Comparison of relative abundance of bacterial phylum in the rumen **(A)**; Comparison of relative abundance of *Alphaproteobacteria* **(B)**.

As shown in Table 4, there were no significant differences in the OTU numbers and the indices of Shannon, Chao1, ACE and Simpson between the two groups. In addition, PCoA also revealed that there was no obvious separation between the two groups (Figure 3). We observed that the dominant phylums of bacteria were *Bacteroidota*, *Firmicutes*, and *Proteobacteria* (Figure 4). Compared with Holstein cattle, *Alphaproteobacteria* were higher in abundance in Montbéliarde×Holstein cattle (*p* < 0.05).

**Table 4:** The alpha diversity of rumen microorganisms in Montbéliarde×Holstein and Holstein Cattle.

Items	H	MH	*p-*value
Observed_species^1^	1664.80 ± 91.48	1954.80 ± 211.01	0.259
Shannon^2^	7.39 ± 0.19	7.85 ± 0.29	0.223
Chao1^3^	1816.21 ± 93.56	2071.64 ± 229.26	0.347
ACE^4^	1837.27 ± 95.67	2099.25 ± 226.58	0.332
Simpson^5^	0.97 ± 0.01	0.98 ± 0.01	0.406

1 Observed_species: the number of visually observed species (that is, the number of OTUs).

2 Shannon: the total number of categories and their proportions in the sample. The higher the community diversity, the more uniform the species distribution, and the larger the Shannon index.

3 Chao1: characterizes the diversity and evenness of species distribution within a community.

4 ACE: Estimate the total number of species contained in the community sample.

5 Simpson: estimate the number of OTUs in the community.

**Figure 5:**
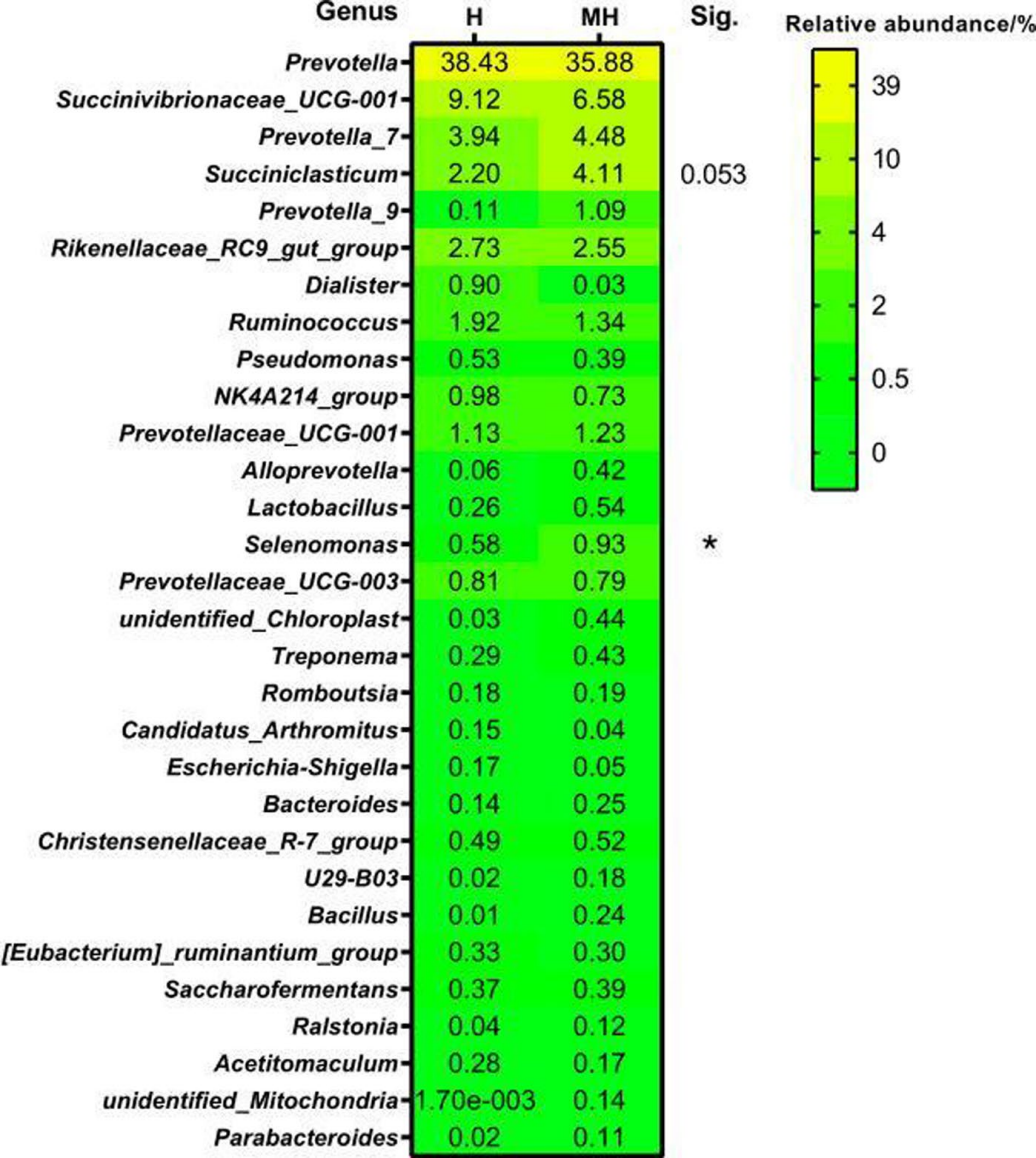
Comparison of genus level of rumen bacteria between Montbéliade×Holstein and Holstein Cattle. The relative abundance of bacteria in the sample is more than 0.5% as the dominant bacterial genus. * indicates significant differences at *p* < 0.05 level.

At the genus level, a total of 572 genera were detected in 10 ruminal fluid samples. Figure 5 shows a comparison of the top 30 genera in relative abundance between the Montbéliarde×Holstein and Holstein cattle. At a relative abundance greater than 0.5%, there were 12 and 13 dominant genera in Holstein and Montbéliarde×Holstein cattle, respectively. There were 10 dominant genera in both groups. In Montbéliarde×Holstein cattle, the abundance of *Selenomonas* and *Succiniclasticum* was higher than that in Holstein cattle (0.93 to 0.58%, 4.11 to 2.20%), though the latter did not reach statistical significance (*p* = 0.053).

### Comparison of ruminal metabolites between Montbéliarde×Holstein and Holstein cattle

**Figure 6:**
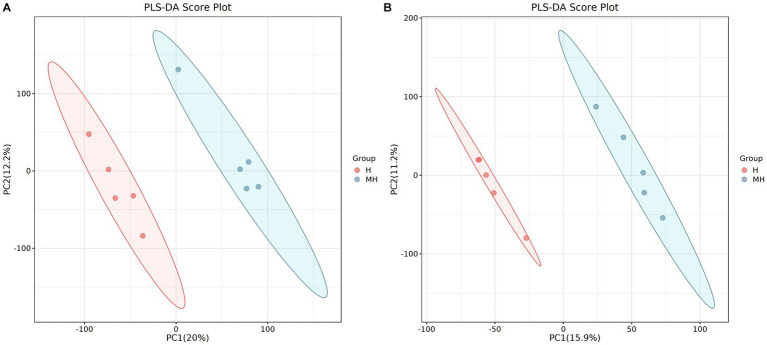
PLS-DA scatter plots of rumen metabolites in the positive ion mode of Montbéliade×Holstein and Holstein Cattle **(A)**; PLS-DA scatter plots of rumen metabolites in the negative ion mode of Montbéliade×Holstein and Holstein Cattle **(B)**.

LC–MS detected a total of 510 metabolites in ruminal fluid. Partial least squares-discriminate analysis (PLS-DA) score plots (Figure 6) of the identified metabolites revealed a clear separation between the two groups. To assess the specific effects of crossbreeding on ruminal metabolites, VIP values obtained from PLS-DA, combined with statistical analysis, were used to screen for differential metabolites. A total of 28 differential ruminal metabolites were detected (VIP > 1.0 and *p* < 0.05), of which 21 were significantly up-regulated and 7 were significantly down-regulated in Montbéliarde×Holstein cattle (Table 5).

**Table 5:** Differential metabolites of Montbéliarde×Holstein and Holstein Cattle.

Metabolites	MH vs. H	Formula	VIP^1^	Rt^2^	Mz^3^
Methylmalonic acid	↑ up	C_4_H_6_O_4_	1.78	833.90	101.07
2-ketobutyric acid	↑ up	C_4_H_6_O_3_	1.88	147.40	102.06
_L_-Serine	↑ up	C_3_H_7_NO_3_	2.05	834.70	105.04
Pyrrole-2- carboxylic acid	↑ up	C_5_H_5_NO_2_	1.78	833.70	111.02
2,3-butanediol	↑ up	C_4_H_10_O_2_S_2_	2.36	261.40	154.99
Imidazol-5-yl- pyruvate	↑ up	C_6_H_6_N_2_O_3_	1.81	323.00	155.05
Isopyridoxal	↑ up	C_8_H_9_NO_3_	2.17	141.30	168.07
Quinaldic acid	↑ up	C_10_H_7_NO_2_	2.10	440.10	174.06
_L_-Homophenylalanine	↑ up	C_10_H_13_NO_2_	1.78	621.30	180.10
_D_-Fructose	↑ up	C_6_H_12_O_6_	1.99	756.70	181.01
Phosphohydroxypyruvic acid	↓ down	C_3_H_5_O_7_P	2.36	560.60	184.99
Glycyl-leucine	↑ up	C_8_H_16_N_2_O_3_	1.93	349.40	189.12
Trans-ferulic acid	↑ up	C_10_H_10_O_4_	1.83	528.20	195.14
O-succinyl-_L_- homoserine	↑ up	C_8_H_13_NO_6_	1.96	143.90	202.07
Capsidiol	↑ up	C_15_H_24_O_2_	1.52	700.40	219.17
Pyrethrosin	↑ up	C_17_H_22_O_5_	1.88	615.40	289.14
11Z-eicosenoic acid	↓ down	C_20_H_38_O_2_	1.72	761.50	311.29
CMP	↑ up	C_9_H_14_N_3_O_8_P	1.80	103.30	324.06
Quinestrol	↑ up	C_25_H_32_O_2_	1.64	613.40	347.24
AMP	↑ up	C_10_H_14_N_5_O_7_P	2.06	140.50	348.07
Lathosterol	↓ down	C_27_H_46_O	2.28	835.80	369.35
Biotinyl-5’-AMP	↑ up	C_20_H_28_N_7_O_9_PS	1.75	322.00	574.13
Lactate	↑ up	C_3_H_6_O_3_	1.72	229.40	90.06
_L_-Norvaline	↓ down	C_5_H_11_NO_2_	2.11	157.30	115.92
Cyanuric acid	↓ down	C_3_H_3_N_3_O_3_	1.78	31.70	128.01
Salicylic acid	↓ down	C_7_H_6_O_3_	1.76	376.10	136.94
Homo-_L_-arginine	↓ down	C_7_H_16_N_4_O_2_	1.61	445.10	187.05
dGMP	↑ up	C_10_H_14_N_5_O_7_P	2.34	105.70	346.06

MH for Montbéliarde×Holstein, H for Holstein. “↑” and “↓” indicate the compounds that were significantly up-regulated and down-regulated in the experimental group relative to the control group, “down” and “up” indicate *p* < 0.05.

1 VIP, Variable important in projection.

2 Rt, Retention time, units.

3 Mz, Mass-to-charge ratio.

### Correlation analysis of differential metabolites with differential microbial genera and milk performance

**Figure 7:**
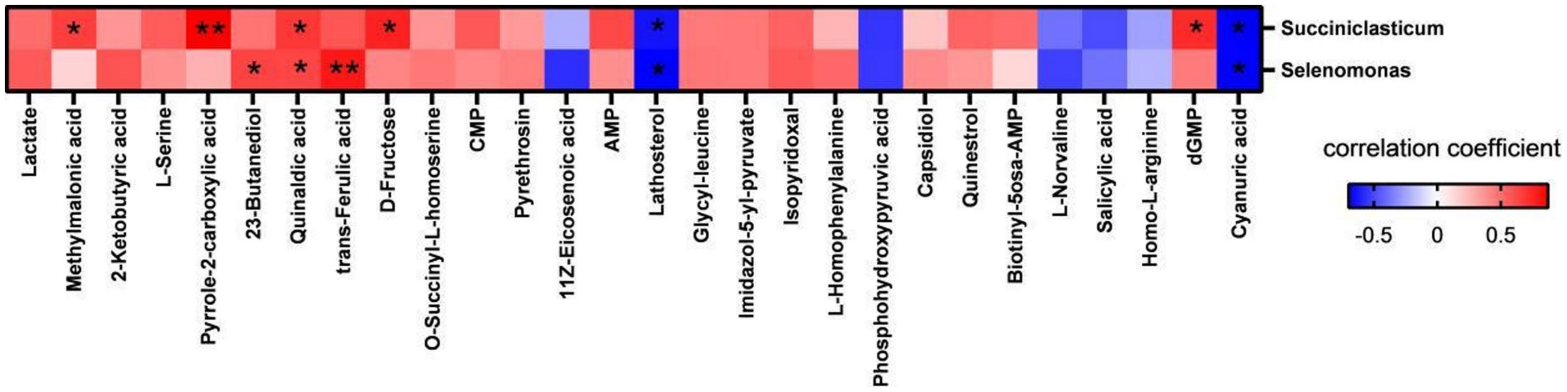
Correlation analysis of significantly changed bacterial genera and metabolites in rumen. Red represents positive correlation, blue represents negative correlation, the darker the color, the higher the correlation; * indicates significant differences at *p* < 0.05 level; ** indicates significant differences at the *p* < 0.01 level.

To explore the interaction between ruminal metabolites and microbiota, a correlation network analysis was conducted using microbial genera and differential metabolites (Figure 7). The results showed that *Succiniclasticum* abundance was positively and significantly correlated with rumen concentrations of methylmalonic acid (*p* = 0.039), quinaldic acid (*p* = 0.030), _D_-fructose (*p* = 0.012), dGMP (*p* = 0.018), and pyrrole-2-carboxylic acid (*p* = 0.001) and negatively and significantly correlated with lathosterol (*p* = 0.042) and cyanuric acid (*p* = 0.024). The abundance of *Selenomonas* showed positive and statistically significant correlations with 2,3-butanediol (*p* = 0.046), quinaldic acid (*p* = 0.049), and trans-ferulic acid (*p* = 0.007), while it was significantly negatively correlated with lathosterol (*p* = 0.025) and cyanuric acid (*p* = 0.024).

**Figure 8:**
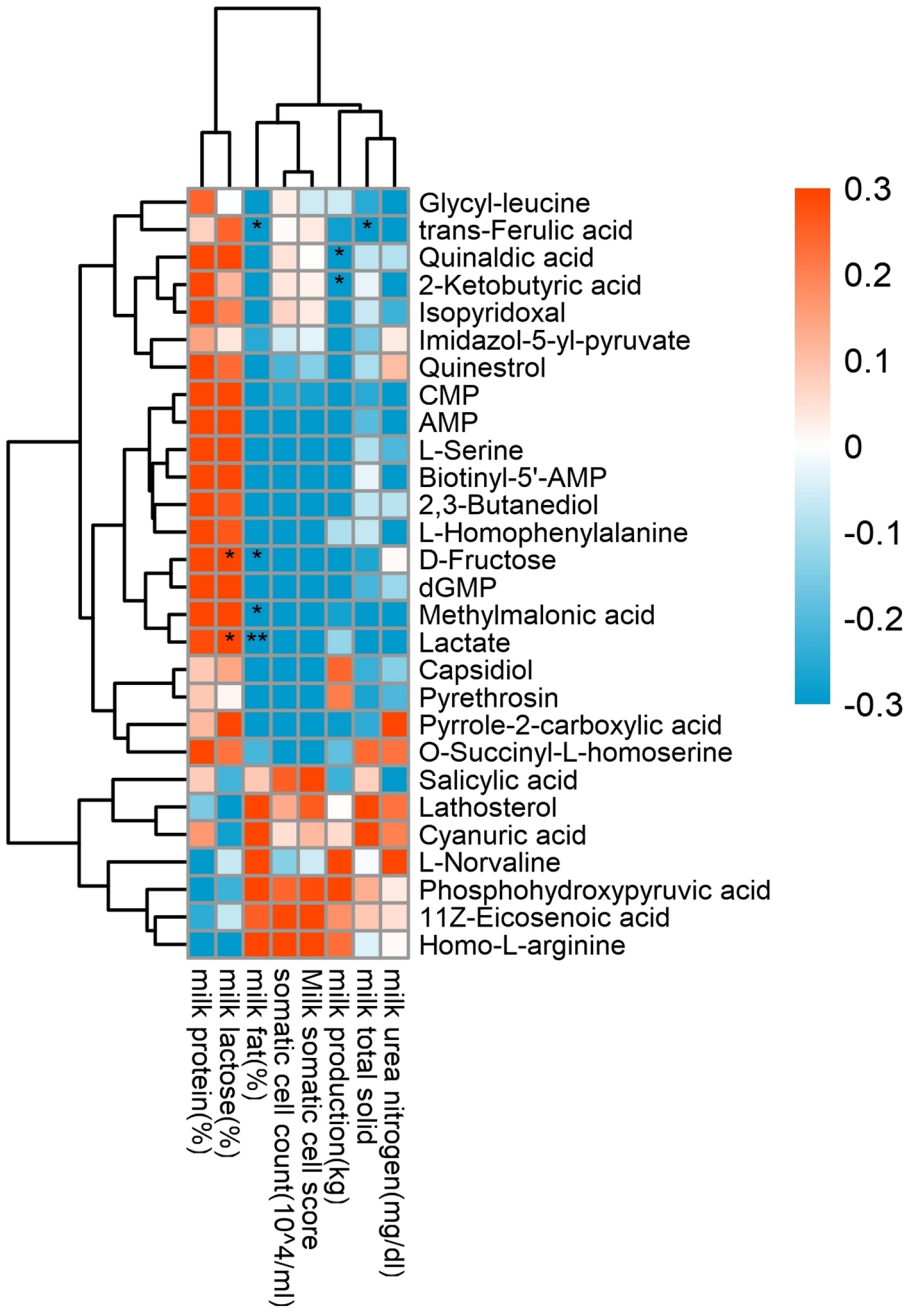
Correlation analysis of rumen differential metabolites and milk performance. Red represents positive correlation, blue represents negative correlation, the darker the color, the higher the correlation; * indicates significant differences at *p* < 0.05 level; ** indicates significant differences at the *p* < 0.01 level.

It can be seen from Figure 8 that milk production (34.00 ± 1.94) was negatively and significantly correlated with 2-ketobutyric acid (*p* = 0.044) and quinaldic acid (*p* = 0.026). Milk fat content (4.87 ± 0.27) was significantly negatively correlated with trans-ferulic acid (*p* = 0.041), _D_-fructose (*p* = 0.025), methylmalonic acid (*p* = 0.012) and lactate (*p* = 0.007). Lactose content (5.60 ± 0.05) was significantly and positively correlated with _D_-fructose (*p* = 0.016) and lactate (*p* = 0.042), and total milk solid rate (16.69 ± 0.21) was significantly negatively correlated with trans-ferulic acid (*p* = 0.040).

## Discussion

While previous studies on Montbéliarde×Holstein cattle have reported on milk performance, they have lacked investigation into ruminal metabolites and microbiota composition. In this study, we compared the differences in milk performance, ruminal microbiota, and ruminal metabolomics between Montbéliarde×Holstein and Holstein cattle and investigated the relationship between ruminal metabolites and both ruminal microbiota composition and milk performance. Our results showed that some indicators of milk performance were better in Montbéliarde×Holstein than in Holstein cattle, and these indicators have significant correlations with different bacteria. The abundance of *Alphaproteobacteria* was significantly increased in Montbéliarde×Holstein compared to Holstein cattle. In contrast, Montbéliarde×Holstein cattle had a greater abundance of *Selenomonas* and *Succiniclasticum* than Holstein cattle, though the latter did not reach statistical significance. A total of 28 differential ruminal metabolites were detected, of which 21 were significantly up-regulated and 7 were significantly down-regulated in Montbéliarde×Holstein cattle. Our data revealed that the first generation of Montbéliarde×Holstein cattle have the characteristics of high milk protein content, low somatic cell score. This provides scientific data support for producing quality dairy products, improving local dairy cattle varieties and the overall efficiency of dairy cattle breeding.

The factors affecting milk production include nutrition and feeding, breeding, environment, disease, cattle comfort etc., among which nutrition and feeding have the greatest influence. Under the same feeding conditions, the average milk production in Montbéliarde×Holstein was 1.76 kg higher than in Holstein cattle, and the average milk production of Montbéliarde×Holstein cattle was higher than that of Holstein cattle from 90 days of lactation, and reached a significant level at 210 days of lactation. This indicated that hybrid cattle may have excellent production performance. Milk protein content is an important indicator of the milk production traits of cattle as well as milk quality (37). Previous studies found that the protein content of milk from Montbéliarde×Holstein cattle was significantly higher than that of Holstein cattle, which was consistent with our results (12). It has also been reported that the fat content of milk from Montbéliarde×Holstein cattle is significantly higher than that of Holstein cattle (12, 13), but our results do not corroborate these findings. The somatic cell count of milk is an important indicator of cattle health and milk quality. Generally, a milk somatic cell count exceeding 400,000 cells/ml indicates that cattle may be suffering from mastitis (38). The number of milk somatic cells in Montbéliarde×Holstein cattle was lower than that of Holstein cattle, though this did not reach statistical significance, indicating that the prevalence of mastitis in Montbéliarde×Holstein cattle may be lower than that of Holstein cattle. The milk somatic cell score of Montbéliarde×Holstein cattle was significantly lower than that of Holstein cattle (1.66 to 2.02), which is consistent with that of Sun (13) and Heins et al. (39) (2.37 to 2.85 and 2.98 to 3.27, respectively). In conclusion, these findings suggest that Montbéliarde×Holstein cattle are less susceptible to mastitis than Holstein cattle.

The rumen is responsible for feed processing in ruminants, capable of digesting 70–85% of the digestible feed and 50% of the crude fiber. Its digestive efficiency depends on the action of the complex microbiota within the rumen. At the phylum level, *Bacteroidota* and *Firmicutes* were the dominant bacterial groups, which together accounted for more than 80% of the total microbial community detected in our study. This is consistent with previous studies in which *Bacteroidota* and *Firmicutes* constitute most microbial communities in cattle at the phylum level, and these bacteria are known to play a role in energy production and metabolism (40). *Alphaproteobacteria*, belonging to the phylum of *Proteobacteria*, were significantly high abundant in the rumen of Montbéliarde×Holstein cattle, compared to that of Holstein cattle. *Proteobacteria* play roles in protein degradation and nitrogen fixation. It is possible that Montbéliarde×Holstein cattle have strong abilities of protein degradation and nitrogen fixation. *Selenomonas* are common bacteria of the rumen which can degrade lactic acid to form acetate and propionic acid and also play an important role in the generation of succinate-propionate (41, 42). In this experiment, the abundance of *Selenomonas* in Montbéliarde×Holstein cattle was significantly higher than that in Holstein cattle, indicating that the utilization of rumen lactic acid may be improved in Montbéliarde×Holstein cattle. However, *Selenomonas* are greatly affected by environmental pH and their rate of lactic acid decomposition is easily inhibited, which can lead to lactic acid accumulation in the rumen (43). This also aligns with our results of the differential ruminal metabolites, which showed that lactate was significantly higher in Montbéliarde×Holstein than Holstein cattle.

Metabolomics allows for a comprehensive understanding of an organism’s physiological and biochemical status. The PLS-DA analysis showed that the ruminal metabolites of Montbéliarde×Holstein cattle were clearly distinguished from that of Holstein cattle. Previous studies have shown that trans-ferulic acid can activate the endogenous antioxidant defense system to eliminate oxygen free radicals (44). Moreover, trans-ferulic acid can regulate transcription factors to produce anticoagulant, antithrombotic and antiplatelet effects, and can regulate cell signal transduction pathways to protect endothelial function (44, 45). Pyrrole-2-carboxylic acid is a common metabolite in many organisms, which has antibacterial, antifungal, anti-inflammatory, and anti-tumor activities (46). Quinaldic acid is a nitrogen-containing heterocyclic compound. Studies have shown that nitrogen-containing heterocyclic derivatives have antibacterial, anti-tumor, antiviral, and anti-inflammatory properties (47). Lathosterol is a cholesterol-like molecule, and it has been shown that excessive accumulation of cholesterol can lead to cellular inflammation and oxidative stress (48). The trans-ferulic acid, pyrrole-2-carboxylic acid, and quinaldic acid concentrations were significantly higher in Montbéliarde×Holstein cattle than in Holstein cattle, while the levels of lathosterol were significantly lower. Thus, it can be speculated that Montbéliarde×Holstein cattle may exhibit greater antioxidant and antibacterial activity.

Through the correlation analysis of differential metabolites with milk performance, we found milk fat content was negatively correlated with methylmalonic acid. Some studies have shown that methylmalonic acid accumulation *in vivo* can inhibit milk fat synthesis (49). The higher levels of methylmalonic acid in Montbéliarde×Holstein compared to Holstein cattle in the current study, which may explain why the fat content of milk from Montbéliarde×Holstein cattle was lower than that of Holstein cattle. _D_-Fructose is a soluble monosaccharide, also known as levulose, which exists in many fruits and honey. Research has shown that fructose reaches complete fermentation after 6 h of incubation under *in vitro* conditions. Microbes in the rumen ferment sugars to produce volatile fatty acids, methane, and carbon dioxide and can also convert monosaccharides into glycogen for storage. These glycogen stores are digested and absorbed by animal tissues, which is one of the sources of glucose in ruminant bodies. 60% of the glucose absorbed by lactating cattle is used to synthesize milk (50). This is consistent with the result that _D_-fructose has a significant positive correlation with lactose content in this experiment. Correlation analysis of differential metabolites with differential microbial genera found that 2,3-butanediol was significantly up-regulated in Montbéliarde×Holstein cattle than in Holstein cattle, and it was also significantly positively correlated with *Selenomonas*. These findings are consistent with previous research showing that 2,3-butanediol is one of the main compounds produced by lactic acid bacteria through the metabolism of acetate (51). *Succiniclasticum* are key constituents of the ruminal microbiota, which mainly use starch as a substrate for fermentation, but can also ferment fiber and cellobiose. *Succiniclasticum* abundance was positively correlated with _D_-fructose, indicating that Montbéliarde×Holstein cattle may have a greater capacity to ferment starch and fiber than Holstein cattle.

In conclusion, this study found that milk protein content from Montbéliarde×Holstein cattle was higher than those of Holstein cattle, while somatic cell scores was significantly lower than those of Holstein cattle. Thus, Montbéliarde×Holstein cattle have the advantage of high milk protein, and the possibility of mastitis is lower than Holstein. Moreover, it can be inferred from the differences in ruminal microbiota composition and metabolomics that Montbéliarde×Holstein cattle may have greater antioxidant capacity, improved antibacterial activity than Holstein cattle.

## Data availability statement

The datasets presented in this study can be found in online repositories. The names of the repository/repositories and accession number(s) can be found below: BioProject, PRJNA946333.

## Ethics statement

The animal study was reviewed and approved by Nanjing Agricultural University Institutional Animal Care and Use Committee. Written informed consent was obtained from the owners for the participation of their animals in this study.

## Author contributions

ZH and HC participated in the study design. HC conducted the experiment, carried out data analysis, and drafted the manuscript. YZ, CW, and NH assisted in sample collection. ZH, HZ, and XW given useful suggestions during writing of the manuscript. ZH directed the study in the whole process. All authors contributed to the article and approved the submitted version.

## Funding

This study was financially supported by the Jiangsu Provincial Breeding of new strains of high-quality dairy cows (JBGS[2021]116).

## Conflict of interest

The authors declare that the research was conducted in the absence of any commercial or financial relationships that could be construed as a potential conflict of interest.

## Publisher’s note

All claims expressed in this article are solely those of the authors and do not necessarily represent those of their affiliated organizations, or those of the publisher, the editors and the reviewers. Any product that may be evaluated in this article, or claim that may be made by its manufacturer, is not guaranteed or endorsed by the publisher.

## Footnotes

^1^http://drive5.com/uparse/

^2^http://www.arb-silva.de/

^3^http://www.drive5.com/muscle/

## Abbreviations

DIM: days in milk
DHI: dairy herd improvement
NMR: nuclear magnetic resonance
LC-MS: liquid chromatography–mass spectrometry
GC–MS: gas chromatography–mass spectrometry
MH: Montbéliarde×Holstein
H: Holstein
TMR: total mixed rations
CTAB: cetyltriethylammnonium bromide
QIIME: Quantitative Microbial Ecological Insights
OTU: operational taxonomic unit
PCoA: Principal coordinate analysis
FWHM: Full width half maximum
HMDB: the human metabolome database
KEGG: kyoto encyclopedia of genes and genomes
LOESS: locally weighted regression
QC: quality control
SCC: somatic cell count
SCS: somatic cell score
RSD: relative standard deviation
PLS-DA: Partial least squares-discriminate analysis
CMP: cytidine monophosphate
AMP: adenosine monophosphate
dGMP: deoxyguanosine monophosphate
